# Evaluation of Caspase-1, Interleukin-1β, and Interleukin-18, in the Middle Ear Effusion in Children With Otitis Media With Effusion

**DOI:** 10.3389/fped.2021.732973

**Published:** 2021-11-05

**Authors:** Shanshan Liu, Lining Guo, Min Chen, Wei Liu, Yanhong Li, Xiaoxu Wang, Shilan Li, Jie Zhang, Xin Ni

**Affiliations:** ^1^Department of Otorhinolaryngology Head and Neck Surgery, Beijing Children's Hospital, Capital Medical University, National Center for Children's Health, Beijing, China; ^2^Beijing Key Laboratory for Pediatric Diseases of Otolaryngology Head and Neck Surgery, Beijing, China

**Keywords:** otitis media with effusion, caspase-1, interleukin-1β, interleukin-18, inflammasomes, children

## Abstract

**Objectives:** The present study aimed to assess the expression of caspase-1 and caspase-1-dependent processing of cytokines, such as interleukin (IL)-1β and IL-18, in the middle ear effusion of children with otitis media with effusion (OME) in order to identify the potential role of inflammasomes in OME.

**Methods:** This study included 29 children scheduled for myringotomy with the insertion of tympanostomy tubes due to OME. Middle ear effusion (MEE) was collected during the surgery. Caspase-1, IL-1β, and IL-18 were assayed using enzyme-linked immunosorbent assay kits. The levels were compared between those with mucoid and serous MEE and those with and without a history of ventilation tube insertion.

**Results:** Caspase-1, IL-1β, and IL-18 were detected in all samples. The caspase-1, IL-1β, and IL-18 levels did not significantly differ between mucoid samples and serous samples. No statistical significances were discovered in caspase-1, IL-1β, and IL-18 levels between with and without a history of ventilation tube groups. There was a significant negative correlation between IL-1β and IL-18 and the duration of OME (*p* < 0.05). However, no significant correlation was found between caspase-1 and disease duration.

**Conclusions:** Inflammasomes may participate in the inflammatory process of OME. IL-1β and IL-18 levels in the MEE decreased over time.

## Introduction

Otitis media with effusion (OME), a common disease in children, is characterized by the collection of fluid in the middle ear without acute inflammatory manifestations (1, 2). This disease is a chronic, low-grade inflammatory condition of the middle ear, and current medication cannot effectively resolve the inflammation. Many inflammatory mediators (such as complement anaphylatoxins, Tumor necrosis factor alpha (TNF-α), interleukin (IL)-1β, and IL-8) and inflammatory cells (such as macrophages, T lymphocytes and B lymphocytes) have been identified in MEE (3, 4). Additionally, it has also been observed that neutrophil extracellular traps (NETs) may play a role in OME (5). However, the molecular mechanisms of the inflammatory condition remain largely unknown. Many studies have shown that IL-1β plays a crucial role in this inflammatory process (6–8). However, IL-1β is produced as an immature form that needs to be cleaved into a mature active form, which is mediated by an inflammasome (9). Furthermore, a study reported linkage between chronic OME and/or recurrent otitis media and chromosome 19q containing several genes involved in the inflammasome protein complex (10). Thus, inflammasomes are suspected to be a key regulator in the pathogenesis of OME.

Inflammasomes are large protein complexes best known for their ability to control activation of pro-caspase-1, and active caspase-1 in turn processes pro-IL-1β and pro-IL-18 into their mature active forms, IL-1β and IL-18, respectively (11). Inappropriate activation of inflammasomes can contribute to the development of various diseases (12). However, the information available regarding the association of inflammasomes with OME is limited. Direct measurement of casepase-1 in pediatric patients with OME has not been performed previously. This is critical to understanding the role of inflammasomes in OME pathogenesis and will also help to advance our understanding of the molecular mechanisms of this disease and the development of novel therapeutic interventions in the future.

Therefore, we aimed to evaluate the expression levels of caspase-1, IL-1β, and IL-18 in the middle ear effusion (MEE) of children with OME in order to explore their clinical significance. To the best of our knowledge, we present the first study concerning MEE Caspase-1 and IL-18 levels in children with OME.

## Materials and Methods

### Study Population

This cross sectional study included children who underwent myringotomy with tube placement for OME our hospital between July 2020 and January 2021. Based on the limited reference (13), a confidence level of 95% was adopted, 20 patients would be adequate to detect IL-1β. OME was diagnosed by otoscopic examination, tympanograms, and hearing assessment according to age. Participants were excluded from the study if they had cleft palate or other craniofacial anomalies, congenital or acquired immunodeficiency, systemic diseases, respiratory tract infection, or received antibiotics within the last month.

Demographic and clinical data, including patient age, sex, medical history, auditory testing results, and type of MEE (mucoid or serous) were recorded. Informed consent was obtained from the parents of each patient for the experimental use of specimens. Approval for this study was granted by the Ethics Review Committee of our hospital.

### Collection, Processing, and Storage of MEE

Prior to surgery, the external auditory canal was washed with 70% alcohol, and a radial incision was made in the anterior inferior quadrant of the tympanic membrane. MEE was collected directly into a 1 mL syringe with the help of a suction device. Each sample was classified as a mucoid or serous MEE based on its appearance and fluidity. Mucoid MEE was a thick and viscous effusion that nearly did not flow when the tube was inverted, while serous MEE was a thin and watery effusion that flowed immediately when the tube was inverted. We found bilateral effusions in one patient who was equally classified. The effusion samples were then transferred to Eppendorf tubes, and bilateral effusions were combined into one tube per patient, and stored at −80°C until analysis.

### Quantification of Caspase-1, IL-1β, and IL-18 Concentrations in MEE

After thawing, sterile saline was added to the samples according to their volume and viscosity, and the samples were then centrifuged at 1300 × g for 10 min. Supernatants from centrifuged samples were collected and diluted to 1:20. Effusions grossly contaminated with blood were excluded from the study. The total protein was measured in each sample using the Bradford Protein Assay Kit (Beyotime Biotechnology, Shanghai, China) following the manufacturer's instructions. The levels of caspase-1 (RayBiotech, Peachtree Corners, Georgia, USA), IL-1β (Abcam, Cambridge, MA, USA), IL-18 (Abcam, Cambridge, MA, USA) were detected using enzyme-linked immunosorbent assay kits according to the manufacturer's instructions.

### Statistical Analyses

Analyses were conducted using SPSS software (version 25.0; IBM, Armonk, NY, USA). The Kolmogorov-Smirnov test was used to assess the normality of the data distribution. The results are expressed as medians and ranges. Statistical differences in the numerical data were analyzed using the Mann-Whitney U-test. The chi-square test was used to analyze categorical data and Linear regression was applied to adjust for covariate. Spearman's correlation was performed to determine correlations. Statistical significance was set at *p* < 0.05.

## Results

### Clinical Characteristics

A total of 29 patients (10 girls and 19 boys) were included in this study. The median age was 55 months (range, 8–180 months), the median disease duration was 12 months (range, 3–36 months), and 8 (27.6%) patients had undergone myringotomy with tube placement before enrollment. The 29 patients in this study were divided into serous and mucoid groups. There were no significant differences between the two groups according to age, sex, duration of OME, and previous ventilation tube insertions (*p* > 0.05) (Table 1). The median concentration of total protein in MEE was 27.52 mg/mL (range, 1.32–34.65 mg/mL). To adjust for the difference in dilutions of samples, the results were corrected as milligrams of total protein. Caspase-1, IL-1β, and IL-18 were detected in all MEE samples (100%), and the levels of caspase-1, IL-1β, and IL-18 were 1520.81 (22.84–53669.4) pg/mg TP, 5.64 (0.01–443.57)pg/mg TP and 578.09 (0.38–2300.79) pg/mg TP, respectively.

**Table 1:** Clinical characteristics of enrolled patients.

**Characteristic**	**Serous group (*n* = 15)**	**Mucoid group (*n* = 14)**	***p*-value**
Male, n (%)	12 (80%)	7 (50%)	0.128
Age, months, median (range)	47 (8–180)	63.5 (12–134)	0.290
Disease duration, months, median (range)	10.5 (3–36)	12 (3–36)	0.910
Previous ventilation tube insertions, n (%)	4 (27%)	4 (29%)	1.000

### Comparison of Caspase-1, IL-1β, and IL-18 Levels Between Serous and Mucoid MEE

**Figure 1:**
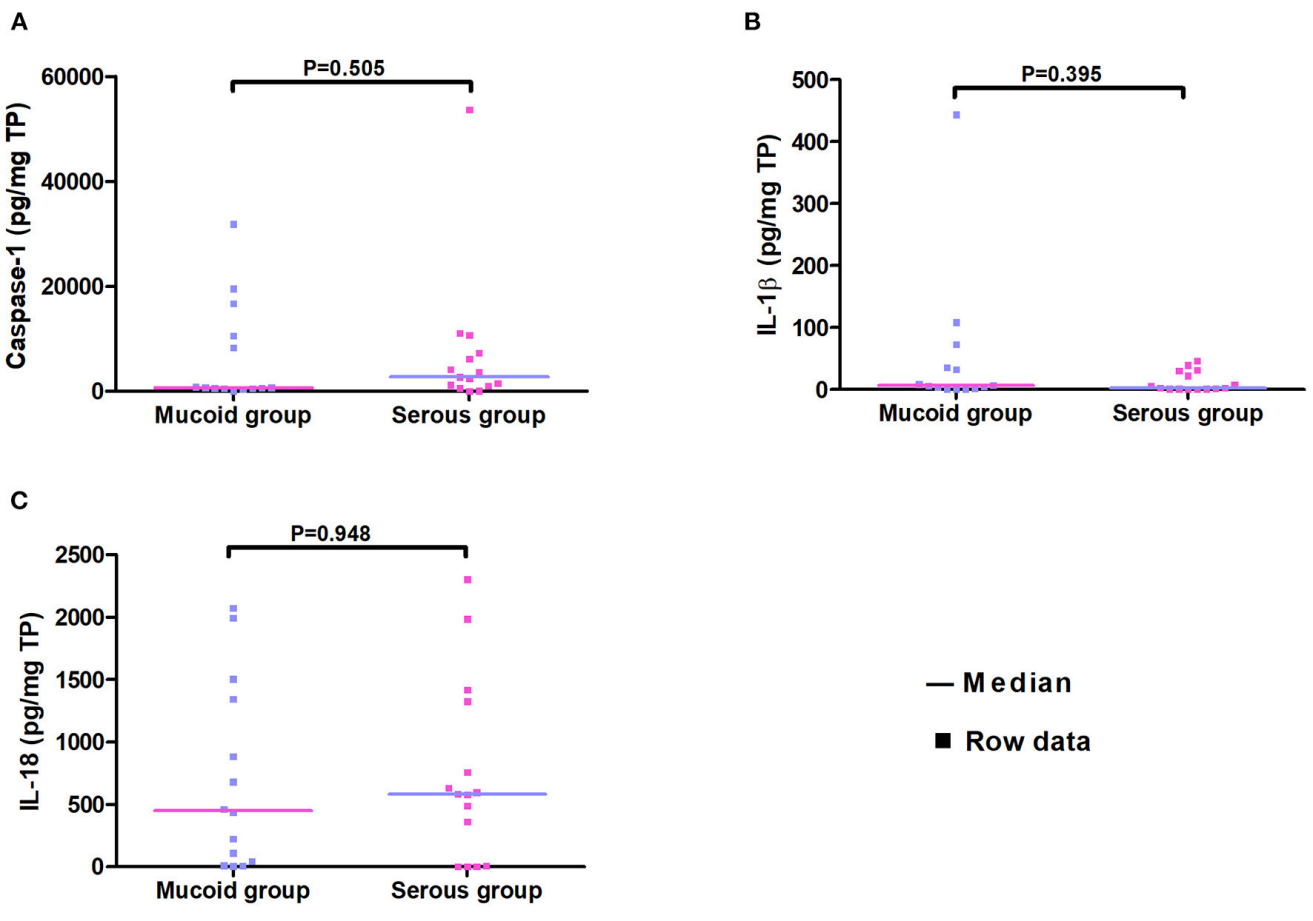
Scatter plot of concentrations of caspase-1, IL-1β, and IL-18 in the serous and the mucoid group. Every dot represents one patient.

Studies have linked serous MEE to better medical outcomes in patients relative to those with mucoid MEE (14, 15). Mucoid and serous MEE have a different composition and biological signatures (16). The concentrations of caspase-1 and cytokines were compared between serous and mucoid MEE. Therefore, the 29 patients in this study were divided into serous and mucoid groups. There were no significant differences between the two groups according to age, sex, duration of OME, and previous ventilation tube insertions (*p* > 0.05). The caspase-1, IL-1β, and IL-18 levels did not significantly differ between mucoid and serous MEE (Figure 1).

### Comparison of Caspase-1, IL-1β, and IL-18 Levels in MEE With and Without a History of Ventilation Tube Insertion

Previous studies have suggested that children who require multiple ventilation tube insertions may have MEE with different inflammatory composition (17). In this study, children were also categorized according to whether they had undergone previous ventilation tube insertion. We found that the levels of caspase-1 and IL-18 were significantly higher in group B (without a history of ventilation tube insertion) than in group A (with a history of ventilation tube insertion) (*p* < 0.05). Between the two groups, the distribution of sex and MEE type did not significantly differ (*p* = 0.39, *p* = 1, respectively). However, the children in group A were significantly younger (49, 8–134 months) than those in group B (88, 37–180 months, *p* = 0.003). The median disease duration was significantly shorter in group A (10.5, 3–36 months) than in group B (18, 8–36 months) (*p* = 0.011). The difference in disease duration between the two groups could be due to the fact that patients who underwent multiple ventilation tube insertions tended to experience longer courses of OME. To adjust for age, linear regression was further performed. As a result, the caspase-1 and IL-18 levels did not significantly differ between groups (Table 2).

**Table 2:** Levels of caspase-1, IL-1β, and IL-18 in the middle ear effusion in patients with and without a history of ventilation tube insertion.

	**Group A (*n* = 21)**	**Group B (*n* = 8)**	***p*-value (Without age adjustment)**	***p*-value (Adjusting for age)**
Caspase-1	3632.11 (456.84–31881.13)	498.405 (22.84–53669.4)	0.024^*^	0.130
IL-1β	6.68 (0.01–443.57)	1.49 (0.18–73.08)	0.218	0.866
IL-18	679.84 (1.29–2300.79)	184.925 (0.38–582.51)	0.011^*^	0.160

*Group A, patients with a history of ventilation tube insertion; Group B, patients without a history of ventilation tube insertion. IL, interleukin. The results are presented as pg/mg total protein, and expressed as the median (range)*.

* *p < 0.05*.

### Correlation of Caspase-1, IL-1β, and IL-18 Levels With Disease Duration

We did not find a correlation between caspase-1 and disease duration. However, we found a significant negative correlation between the cytokine concentrations and disease duration (*p* < 0.05) (Table 3). Because MEE was discovered during an outpatient visit due to sleep snoring in one patient, the exact duration of MEE could not be determined in this patient. Therefore, 28 patients were included in the analysis. The clinical indication for tympanostomy tube placement was MEE persisting for ≥3 months; thus, the minimum duration of OME in this study was 3 months.

**Table 3:** Spearman correlation analysis between levels of caspase-1, IL-1β, and IL-18 in the middle ear effusion and the duration of otitis media with effusion.

	**caspase-1**		**IL-1β**		**IL-18**	
	***r***	***p***	***r***	***p***	***r***	***p***
Disease duration	−0.205	0.296	−0.417	0.027^*^	−0.552	0.002^**^

*Subject number was 28. IL, interleukin*.

* *p < 0.05*,

** *p < 0.01*.

## Discussion

The canonical inflammasome complex contains a nucleotide-binding domain and leucine-rich-repeat-containing [NLR] protein or an AIM2-like receptor [ALR] protein, apoptosis-associated speck-like protein containing a CARD [ASC], and pro-caspase-1 (11). Regarding the potential role of inflammasomes in middle ear diseases, one experimental animal study revealed aberrant overexpression of NLRP3, ASC, caspase-1, and IL-1β in lipopolysaccharide-induced otitis media (18). In another experimental study, lower inflammatory responses were found in ASC-deficient mice with acute otitis media (19). Furthermore, the levels of NLRP3, ASC, and caspase-1 mRNA were significantly upregulated in middle ear tissue samples from patients with cholesteatoma or chronic otitis media (20). Inflammasomes are intracellular platforms of proteins and activated Caspase-1 is secreted alongside mature cytokine after inflammasome activation (9). Therefore, in this study, we measured the protein expression of caspase-1 in the MEE of pediatric patients with OME and found that caspase-1 were detected in all MEE samples (100%). This result suggests that inflammasomes could be involved in the pathogenesis of OME.

One study regarding the effects of IL-1β on Na(+)-K(+)-2Cl(-) cotransporter (NKCC) in human middle ear cells suggested that IL-1β upregulates NKCC1 in middle ear epithelial cells, which is one of the important underlying mechanisms of excess fluid collection in OME (7). IL-1β can reduce middle ear epithelial fluid absorption via the sodium channel (ENaC) by suppressing the ENaC-dependent current, thus contributing to fluid retention in OME (21). Moreover, IL-1β seems to be related to dysfunction of the Eustachian tube (8). Together, these findings indicate that IL-1β plays an important role in the pathogenesis of OME. IL-1β has been detected many times in the MEE of patients with OME. In this study, we found that IL-1β was expressed in all MEE samples from pediatric patients with OME (100%). Several early studies also demonstrated that IL-1β is expressed in most MEE samples in children with OME (17, 22, 23). In contrast, Yellon et al. (24) reported that IL-1β was only detected in 58% of MEE samples. However, to the best of our knowledge, few studies have explored the role of IL-18 in OME. Our findings showed that IL-18 expression was detected in all MEE samples, and IL-18 concentration was 100-fold higher than that of IL-1β. Therefore, it is necessary to explore the role of IL-18 in the pathogenesis of OME.

It seems that mucoid and serous MEE have different compositions and biological signatures (16). Remodeling of the epithelium of the middle ear is needed for the production of mucoid MEE, which indicates that patients with mucoid effusion would experience heavier remodeling than patients with serous MEE (25). Therefore, mucoid MEE may represent a stronger inflammatory response in the middle ear. To determine whether the levels of inflammatory proteins, caspase-1, IL-1β, and IL-18 differ between serous and mucoid MEE, we divided all patients into serous and mucoid groups according to the MEE type. However, we found that the caspase-1, IL-1β, and IL-18 levels did not significantly differ between mucoid group and serous group. Early studies reported that IL-1β levels in mucoid MEE were significantly higher in mucoid MEE than in serous MEE (26, 27)). Our results showed that expression levels IL-1β did not differ significantly between the two groups. In accordance with our results, Yellon et al. (28) found no significant difference in the levels of IL-1β with the type of effusion.

IL-1β and IL-18 both belong to the proinflammatory IL-1 family (29). They are induced at the earliest stages of the innate immune response and stimulate a subsequent cascade of proinflammatory cytokines (30). In our study, we found that both cytokines were significantly negatively correlated with disease duration. Himi et al. reported that IL-1β was significantly associated with neutrophil infiltration in the middle ear space (31). Thus, the decrease in IL-1β over time may be associated with the infiltration of different inflammatory cell types during the development of this disease. Inconsistent with this result, Skotnicka et al. (22) found no correlation between the IL-1β level and the duration of OME. The imbalance of T helper (Th)1/Th2, toward the Th2 response, is considered to be involved in the pathogenesis of OME (6, 32). IL-18, a proinflammatory cytokine, facilitates the Th1 response by inducing interferon-γ production by Th1 cells (33). The downregulation of the Th1 cytokine IL-18 may contribute to the maintenance of chronic inflammation in the middle ear.

Early studies suggested that the concentrations of tumor necrosis factor-α and C3 were higher in MEE samples from children who had undergone multiple ventilation tube insertions (17, 28). In this study, we aimed to evaluate the levels of caspase-1, IL-1β, and IL-18, in patients with and without a history of ventilation tube insertion. Without age adjustment, we found that the caspase-1 and IL-18 levels were significantly higher in patients without a history of ventilation tube insertion than in those with a history of ventilation tube insertion. However, after adjusting age, the significant differences disappeared. This finding suggested that the differences might be age dependent. Similarly, a previous study reported that the expression of cytokine mRNAs in the MEE of children with OME differed by age, which may explain why susceptibility and treatment outcomes of OME vary in different age groups (34).

This is the first study to evaluate the level of caspase-1 in MEE samples from children with OME and investigate the correlation between caspase-1 and clinical parameters. The results of this study indicate that inflammasomes may participate in the pathogenesis of OME. This study had several limitations. First, the collection of mucoid MEE was difficult, and samples were easily contaminated by blood during collection; therefore, the sample size was small. Second, there was no effusion in a healthy middle ear cavity; hence, we did not set up a control group. Third, we only measured the level of protein and lacked the measurement of mRNA. We recommend further studies with larger sample sizes and diverse detection methods to confirm the validity of our findings.

## Data Availability Statement

The original contributions presented in the study are included in the article/supplementary material, further inquiries can be directed to the corresponding author/s.

## Ethics Statement

The studies involving human participants were reviewed and approved by Ethics Review Committee of Beijing Childern's Hospital. Written informed consent to participate in this study was provided by the participants' legal guardian/next of kin.

## Author Contributions

SLiu, JZ, and XN: conceptualization. SLiu, LG, YL, XW, and SLi: data curation. SLiu and LG: formal analysis. JZ and XN: funding acquisition and writing–review and editing. MC, WL, JZ, and XN: methodology. SLiu: project administration, visualization, and writing–original draft. All authors contributed to the article and approved the submitted version.

## Funding

This work was supported by Pediatric Medical Coordinated Development Center of Beijing Hospitals Authority (XTYB201828) and Beijing Hospitals Authority′ Ascent Plan (DFL20191201).

## Conflict of Interest

The authors declare that the research was conducted in the absence of any commercial or financial relationships that could be construed as a potential conflict of interest.

## Publisher's Note

All claims expressed in this article are solely those of the authors and do not necessarily represent those of their affiliated organizations, or those of the publisher, the editors and the reviewers. Any product that may be evaluated in this article, or claim that may be made by its manufacturer, is not guaranteed or endorsed by the publisher.
